# Epilepsy and noncompaction

**DOI:** 10.1007/s12471-013-0497-5

**Published:** 2013-11-21

**Authors:** J. Finsterer, S. Zarrouk-Mahjoub

**Affiliations:** 1 Krankenanstalt Rudolfstiftung, Postfach 20, 1180 Vienna, Austria; 2 Laboratory of Biochemistry, UR “Human Nutrition and Metabolic Disorders” Faculty of Medicine Monastir, Monastir, Tunisie

**Keywords:** Non-compaction, Left ventricular hypertrabeculation, Cardiomyopathy, Syncope, Arrhythmia, Epilepsy, Seizures

Dear Sir,

With interest we read the article by Dello et al. about a patient with left ventricular hypertrabeculation/noncompaction (LVHT) and syncope being attributed to ventricular tachycardia [1]. We have the following comments and concerns.

Work-up for syncope not only requires cardiological but also neurological investigations. It is important not only to rule out epilepsy but also ischaemic stroke or cerebral bleeding. In this respect it is important to carry out multimodal cerebral MRI to receive not only parenchymatous but also vascular information. Stroke is particularly prevalent in LVHT patients because of embolism from thrombus formation within the intertrabecular spaces [2]. Single patients with LVHT may also develop atrial fibrillation, the most common risk factor for embolic stroke. A number of patients with LVHT also develop systolic dysfunction, another risk factor for stroke or embolism [3]. Did the described patient undergo cerebral MRI and what was the result? Where any old ischaemic lesions seen on TIRM sequences?

Syncope may also derive from stenosis of the extra-cranial cerebral arteries. Did the patient undergo carotid ultrasound or contrast-enhanced MR angiography to rule out high-grade stenosis of the internal carotid arteries or dissection of the carotid or vertebral arteries?

A further neurological cause of syncope may be affection of the peripheral nerves [4]. Were there any indications for vegetative polyneuropathy in the described patient? Did he present with weakness, wasting, reduced tendon reflexes, or sensory disturbances? Was the patient seen by a neurologist to rule out or confirm acute or chronic polyneuropathy?

A fourth reason why the neurologist should see LVHT patients is the frequent association of LVHT with neuromuscular disorders [5]. Particularly muscle disease is highly prevalent among LVHT patients, although a causative relation between neuromuscular disorders and LVHT has not been proven yet.

It would be also interesting to receive a more detailed description of the paroxysmal activity on EEG? How often did the patient experience seizures? What type of seizures did he experience? Only focal or also generalised seizures? Did he also present with non-convulsive seizures? When was the onset of epilepsy, what was the seizure frequency, what were the most frequent triggers for seizures in this particular patient? Did he have a family history positive for epilepsy, did he have a history of birth trauma, cerebral hypoxia, fever cramps, traumatic brain injury, meningitis, or a history of neurosurgery? What was the cause of epilepsy and did he ever take antiepileptic drugs other than levetiracetam? How do the authors know that paroxysmal activity on EEG was secondary to arrhythmias and not primary due to a double trouble? It is not unusual that EEG is normal after a seizure, particularly after initiation of antiepileptic treatment. EEGs may be also abnormal in patients without epilepsy, such as in patients with migraine or other types of headache. Did the patient report a history of headache? Did he experience further seizures after discharge? How frequently did he attend the follow-ups? How were the follow-up EEGs? Was the family history positive for epilepsy?

Overall, there is a strong need to investigate LVHT patients with a history of syncope or epilepsy more thoroughly, particularly by referral to the neurologist. The neurologist must rule out stroke, epilepsy, carotid artery stenosis, polyneuropathy, or myopathy. Seizures in LVHT patients may not only be due to ischaemia during ventricular arrhythmias but also an independent second trouble in these patients.
